# Verification of the Mediating Effect of Social Support on Physical Activity and Aging Anxiety of Korean Pre-Older Adults

**DOI:** 10.3390/ijerph17218069

**Published:** 2020-11-02

**Authors:** Ahra Oh, Jiyoun Kim, Eunsurk Yi, Jongseob Shin

**Affiliations:** 1 Exercise Rehabilitation Convergence Institute, Gachon University 191 Hombakmoero, Yeonsu-gu, Incheon 406-799, Korea; oh-yang0329@hanmail.net; 2 Department of Exercise Rehabilitation & Welfare, Gachon University 191 Hombakmoero, Yeonsu-gu, Incheon 406-799, Korea; yies@gachon.ac.kr

**Keywords:** pre-older adults, social support, physical activity, aging anxiety, Korea

## Abstract

There is a lack of research on Korean prospective elderly persons. In particular, there is little research regarding whether social support has a mediating effect on the relationship between physical activity and aging anxiety. Accordingly, this study investigated how social support affected physical activity and aging anxiety in 778 prospective senior citizens (55 to 65 years old) out of a total of 1447 senior citizens who participated in the Embrain Panel Power and Panel Marketing Interactive. Participants completed the IPAQ (International Physical Activity Questionnaires), Social Support Scale, and Aging Anxiety Scale. Physical activity in these Korean pre-older adults affected aging anxiety (*p* < 0.001), with a fixed effect of physical activity on social support (*p* < 0.001). Further, social support affected aging anxiety (*p* < 0.001). Social support was also an important parameter in the relationship between physical activity and aging anxiety. In conclusion, high physical activity of pre-older Korean persons lowered their anxiety regarding aging. Social support acted as a mediator that lowered anxiety regarding aging in the most active pre-older persons.

## 1. Introduction

In 2019, the Korean Legislative Research Office published a report on the lives of Korean older adults according to the Organization for Economic Co-operation and Development (OECD) statistics [1]. According to this report, the characteristics of Korean older adults may be distinguished from those of older adults in other OECD member countries. The report indicated that Korean senior citizens participate in irregular low-wage labor to pay for their livelihood even after retirement, and the proportion of senior citizens aged 65 or older is one of the largest among OECD member countries. In addition, the poverty rate was ranked first among OECD members and subjective health was ranked 30th out of 35 OECD member countries, with most senior citizens reporting poor subjective health. The most serious issue is the fact that Korean older adults are very socially isolated, and the suicide rate is also the highest among the member countries. This indicates that the Korean government is not properly implementing health promotion programs, welfare programs, and care for the elderly. Above all, that children do not take care of their parents represents a harsh aspect of Korean society.

The difficulties these older adults are experiencing potentially relates to the Korean War of the 1950s, during which many lost their homes. Families were separated and there was a shortage of labor. Their coping strategy was predominately to diligently save money to develop their families and their country. Therefore, they have been working all their lives. It has become a habit to live as carefully as possible, since it appears to them the only way to ensure the safety of their family. Namely, it has become a habit to live with utmost care, and to devote oneself to caring for one’s children at the expense of one’s own needs [2]. Because of such practices, the Korean economy grew rapidly in the 1960s [3], and Korea is now one of the most economically advanced countries worldwide.

In contrast, the post-Korean War generation have observed elderly persons experiencing difficulties. The post-Korean War generation typically experienced a difficult time in childhood, but thrived in later life. They wished to live a leisurely life because they observed the difficulties their parents experienced. The post-Korean War generation is called the older generation in Korean society, and there is a notable difference between the thought patterns and cultural aspects of this group and those of their parents. Those aged 55 to 65 years are referred to as pre-older adults [4], or active seniors [5].

Korean pre-older adults observed the difficult lives of their parents. Accordingly, pre-older people do not want to experience the difficulties faced by their parents. Thus, pre-older people tend to have completely opposite lifestyles than their parents. Pre-older persons are familiar with the information technology environment and freely consume technological products. Primarily, pre-older adults thoroughly prepared for a healthy retirement due to deep anxiety about their aging [5]. Most also chose active lives, investing in themselves to overcome aging-related issues. Moreover, pre-older persons actively participate not only in consumption, but also in various cultural and social activities [3,6,7].

In this sense, the lives of pre-older adults in Korea significantly differ from those of the current older adults, especially in the engagement of the former in social activities. Various social activities are of paramount importance to older adults not only to enhance the body’s immunity and health, but also to create a sense of psychological stability and open-mindedness [8]. In particular, social activities that involve effective communication and that expand social groups can lead to larger support structures and a greater presence of older adults in society [8,9]. This represents social support, namely support received from family, friends, and neighbors [10]. People with high availability of social support can readily receive assistance in a positive environment.

Engaging positively with the surrounding environment relates to positive thoughts and confidence in others [11,12,13]. Self-confident people are more energetic and physically active than those who lack self-confidence [11,12]. This action can draw support from others [14,15,16]. That is, people who engage in vigorous physical activity are less likely to be affected by psychological anxiety and are more likely to overcome this state [17]. In contrast, people with less social support may experience strong psychological anxiety, since they lack support, which increases fear, psychological atrophy, and tension.

In addition, aging anxiety is a psychological concept that refers to anxiety and fear about becoming an older adult. This is a complex concept that encompasses the loss of power over one’s body, psychology, and society. Addressing aging anxiety is an important key to adaptation to aging [18,19]. Being physically active increases social support, and high social support helps one to accomplish more. Anxiety has the converse affect, but since aging anxiety is a psychological issue, if physical activity and social support are high, aging-related anxiety can be expected to have a low effect on the individual.

Positive effects of physical activity have been found in previous research. Studies have shown that high physical activity and social support negatively affect aging anxiety, while social support increases the amount of physical activity and lowers aging anxiety. Therefore, it is very likely that social support plays a very important mediating role [20,21,22]. However, studies have not detailed how social support affects the physical activity of older Korean adults. Further, no study has investigated the impact of psychosocial variables on aging anxiety.

Thus, there is a need to explore how social support and beliefs about aging impact the pre-older adult group in Korea.

Therefore, this study investigated how social support affects physical activity and aging anxiety in Korean pre-older adults. The following hypotheses were proposed. First, the amount of physical activity will have a negative (−) effect on aging anxiety. Second, the amount of physical activity will have a positive (+) effect on social support. Third, social support will have a negative (−) effect on aging anxiety. Fourth, social support will mediate the relationship between physical activity and aging anxiety.

### 1.1. The Relationship Between Physical Activity, Aging Anxiety, and Social Support

The relationship between the amount of physical activity and social support shows that humans are more likely to pursue a healthy lifestyle when social support increases [23,24]. Social support refers to support received from others. Therefore, the availability of social support implies living a more vibrant life and feeling recognized by others as a valued member of a community [25]. In addition, social support is related to psychological concepts such as depression, stress, and confidence [26,27]. The observations apply equally to pre-older adults, such that when pre-older adults have available social support both inside and outside the home, a more positive and vibrant life is possible. Studies have indicated that social support is a mediating variable that can influence the amount of physical activity pursued [28,29].

The relationship between aging anxiety and social support was found to be less affected by emotions and stress when social support was high [30,31]. From one perspective, weak social support is likely to lower morale as well as social activity by creating a lack of confidence and promoting anxiety [30,31]. Aging anxiety is also a psychological state; as such, these results should also apply to pre-older adults. High social support can positively influence aging and engender positive aging without depression or stress [32,33]. Thus, we conclude that social support is a mediator that has a negative effect on aging anxiety.

Finally, the relationship between physical activity and aging anxiety decreases anxiety as physical activity increases; these results improve health [34,35,36]. Therefore, people who engage in considerable physical activity usually have less anxiety and less stress than less-active people. Older adults are at a life-stage that is rapidly affected by aging-related psychological changes. Even physically healthy older adults experience psychological change [37]. However, anxiety increases when physical activity is low [38,39]. That is, there is a negative relationship between aging anxiety and physical activity among pre-older adults [40,41,42,43].

### 1.2. Hypotheses and Research Framework

**Figure 1:**
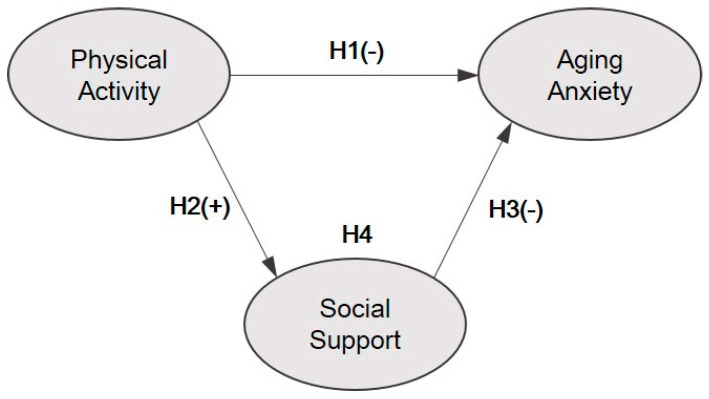
A conceptual model of the relationship between physical activity, social support, and aging anxiety among pre-older adults in Korea.

The hypotheses of this study were established based on the relationship between physical activity and aging anxiety, the relationship between physical activity and social support, and the relationship between social support and aging anxiety. The model of the hypotheses is shown in Figure 1.

The hypotheses corresponding to each path in the conceptual model were as follows.

**Hypothesis 1 (H1).**  *Physical activity will have a negative (−) effect on aging anxiety.*

**Hypothesis 2 (H2).**  *Physical activity will have a positive (+) effect on social support.*

**Hypothesis 3 (H3).**  *Social support will have a negative (−) effect on aging anxiety.*

**Hypothesis 4 (H4).**  *Social support will mediate the relationship between physical activity and aging anxiety.*

## 2. Method

### 2.1. Participants and Procedure

A questionnaire was commissioned from the Embrain Panel Power (https://www.panel.co.kr/) and Panel Marketing Interactive (http://www.pmirnc.com/), which are Korean survey agencies. A survey was conducted with people aged 50 to 85 years in Korea.

To better explain the differences between older adults and pre-older adults in Korea, the research team asked the survey agency to equally select older adults and pre-older adults in large cities and small and medium-sized cities in all regions of Korea. The entire survey process was conducted by telephone, for which participants received KRW 3900. Of the 1447 respondents, 778 pre-older adults (55–65 years old) were selected as participants. The sample size was calculated using the G-Power 3.1 program. Effect size, α level, test power, and number of predictors were set to 0.15, 0.05, 0.95, and 2, respectively; as such, the minimum number of participants in this study was 107, with 778 participants actually recruited. The experimental procedure was performed in accordance with the Ethical Committee of Gachon University, which approved the study (1044396-202007-HR-124-01).

The total number of study participants was 778, of whom 433 were male (55.7%) and 345 were female (44.3%). There were 261 participants aged 55–59 years (33.5%) and 517 participants aged 60–65 years (66.5%); 385 (49.5%) lived in larger cities and 392 (50.5%) in small and medium cities.

### 2.2. Measurements

#### 2.2.1. Physical Activity

To assess physical activity, the Korean version of the International Physical Activity Questionnaires (IPAQ) officially recognized by the IPAQ Development Team was used [44]. The content includes the number of days and average hours of intense activity, moderate physical activity, and walking activity performed for 10 min or longer. Participants were also asked how many hours they spent sitting down. Subsequently, these data were calculated as Metabolic Equivalent Task (MET)-minutes scores based on the IPAQ score conversion method. The calculation formulae are shown in Table 1.

In addition, physical activity was classified into three groups according to the international physical capacity evaluation scoring system. Low-intensity physical activity included persons who did not report any activity. The moderate physical activity group reported at least 20 min of intense activity per day for three days or more, at least 30 min of moderate activity or walking for five days or more, or, a combination of walking, moderate physical activity, and vigorous physical activity for at least 600 MET-min/week for five days or more. The high-intensity physical activity group included those active more than 1500 MET-min/week through intense activity for more than three days a week, or more than 3000 MET-min/week through complex physical activity of walking for seven days or more, moderate activity, and intense activity (only one participant met these criteria).

#### 2.2.2. Social Support

Social support was assessed using a scale translated by Jung and Chun [45] from the Social Support Scale developed by Zimet et al. [10]. The scale consists of four questions, each of which addresses support from family, from friends, and from significant others, making a total of 12 items. These twelve items were rated on a 5-point Likert scale ranging from “not at all” (1 point) to “very much” (5 points); the higher the score, the higher the social support. Cronbach’s α was 0.95 in Jung and Chun [45]. This questionnaire was used for older adults who had recently retired [46].

#### 2.2.3. Aging Anxiety

The Anxiety about Aging Scale developed by Lasher and Faulkender [19] was used as an aging anxiety measurement tool, in the form as modified by Lee and You [47] for middle-aged women. The scale consists of 20 questions that address four factors: “Fear of Loss” (4 questions), “Physical Appearance” (4 questions), “Fear of Old People” (5 questions), and “Psychological Concerns” (7 questions). Responses range from “Not at all” (1 point) to ”Very much” (5 points); the higher the score, the higher the aging anxiety. Cronbach’s α was 0.90 in Lee and You [47]. This questionnaire has been recently used for elderly Koreans [48].

### 2.3. Design and Data Analysis

SPSS 22.0 (IBM Corp., Armonk, NY, USA) was used to perform descriptive statistical analysis, exploratory factor analysis, reliability analysis, confirmatory factor analysis, and correlation analysis. Then, AMOS 25 (IBM Corp., Armonk, NY, USA) was used to conduct and verify structural equation modeling.

Exploratory factor analysis was conducted to verify scale validity. For social support, three factors were extracted from 12 questions. Cronbach’s *α* for social support indicated good internal consistency reliability (*α* = 0.925–0.955). The results are shown in Table 2.

For aging anxiety, four factors were extracted as a result of factor analysis of 20 items, excluding two questions with low factor loading values. Cronbach’s *α* values for aging anxiety were 0.764–0.851. The results are shown in Table 3.

Confirmatory factor analysis was performed to derive a measurement model for each variable verified through exploratory factor analysis and a reliability analysis. The recommended Tucker–Lewis index (TLI), comparative fit index (CFI), and root mean square error of approximation (RMSEA) indices were used to assess the goodness-of-fit of the measurement model in consideration of model fit and simplicity [49]. The reference value of the indices was interpreted as an acceptable fit, namely TLI and CFI > 0.900 and RMSEA < 0.050–1.000 [50]. In addition, it is recommended that χ^2^ and df be presented in the study results. This is because these two statistics are the basis for almost all other indices [51].

The χ^2^ (df = 51) value for social support was 437.954, TLI was 0.949, CFI was 0.961, and RMSEA was 0.099. The χ^2^ (df = 98) value for aging anxiety was 404.788, TLI was 0.924, CFI was 0.938, and RMSEA was 0.630. Therefore, the model had acceptable fit. In addition, concept reliability (CR) and average variance extracted (AVE) were calculated to verify the centralized validity between each measurement variable. AVE and CR values for social support and aging anxiety are shown in Table 4 and Table 5.

Before analyzing the relationship between physical activity, social support, and aging anxiety, the Pearson correlation coefficient between each variable was analyzed. Table 6 shows the correlations between the factors, which confirmed the discriminant validity between the factors for which single-dimensionality was confirmed.

As shown in Table 6, since no correlation coefficient exceeded 0.85, discriminant validity was present according to the criteria of Kline [52].

## 3. Results

The model of this study encompassed results from three scales: physical activity, social support, and aging anxiety. To verify the mediating effect hypothesized in this study, an overall effect model and a mediating effect model were tested. Accordingly, a structural spinning model was developed. In particular, in the mediating effect model, the effects of the independent variable on the dependent variable were divided into a direct effect, indirect effect, and total effect, consisting of the combination of direct and indirect effects.

The four criteria for evaluating mediating effects as proposed by Baron and Kenny [53] were adopted. First, the overall effect should be statistically significant. Second, the influence of independent variables on parameters should be established. Third, the influence of parameters on dependent variables should be noted. Fourth, a mediating effect exists when the influence of the independent variable on the dependent variable is not statistically significant.

The first stage of Baron and Kenny’s evaluation of mediating effects [53] can be confirmed via the overall model effect in structural equation modeling. In the absence of social support, physical activity and the path to aging anxiety were analyzed. The fit of the overall effect model was as follows: χ^2^ = 63.891 (df = 5), incremental fit index (IFI) = 0.912, normed fit index (NFI) = 0.905, CFI = 0.911, and RMSEA = 0.123. RMSEA < 0.100 denotes moderate fit; although RMSEA exceeded 0.100, the incremental measures IFI, NFI, and CFI all showed good fit (all > 0.900). The path coefficient of the overall effect model is shown in Table 7.

In the absence of social support as a mediating variable, physical activity had a significant negative effect on aging anxiety. As suggested by Baron and Kenny [53], the influence of independent variables on parameters should be noted. To assess the influence of the parameter on the dependent variable, a parameterization effect model was analyzed, whose fit statistics were as follows: χ^2^ = 139.802 (df = 18), IFI = 0.938, NFI = 0.930, CFI = 0.938, and RMSEA = 0.093, indicating satisfactory overall fit. Therefore, the mediating model was suitable; the corresponding path coefficients are shown in Table 8.

Physical activity had a significant negative effect on aging anxiety, confirming H1. The independent variable, physical activity, had a significant positive effect on social support, supporting H2. H3 was supported by the finding that social support as a mediating variable had a significant negative effect on aging anxiety as a dependent variable. Accordingly, a mediating effect was present on social support. The total effect, direct effect, and indirect effect in the path analysis are shown in Table 9.

To verify the statistical significance of the mediating effect and the indirect effect, bootstrapping was conducted using AMOS. Specifically, in the validation of mediating effects, the null hypothesis that there was no mediating effect was assessed by using bootstrapping to generate the applicable confidence interval [54] (Table 10).

In Table 5, the 95% confidence interval (CI) was −0.171 to −0.078. Since zero was not included in the interval, the null hypothesis was rejected and research hypothesis H4 supported. To specifically verify the mediating effect of social support, a partial mediation model and a complete mediation model were introduced as competitor models. To distinguish between the two competitor models, statistical significance of the χ^2^ difference was assessed; the fits of the structural equation models were compared (Table 11).

In the partially mediated model, the df decreased by 1 and the χ^2^ value decreased by 5.499 compared to the fully mediated model. Since the critical values of χ^2^ (df = 1) for *p* < 0.05 is 3.84, the two models were statistically different. In addition, TLI was 0.905 in the partially mediated model and 0.904 in the fully mediated model; fit of the partial mediated model improved. RMSEA suggested decreased fit in the partially mediated model (0.092) versus the fully mediated model (0.093). Given the fit results, the fully mediated model was more suitable than the partially mediated model. Therefore, the fully mediated model was finally adopted.

## 4. Discussion

First, physical activity had a negative (−) effect on aging anxiety. Greater physical activity improves health status compared to persons who do not engage in such activity [55,56,57]. In addition, healthy people tend to exert control over their lives [58,59] and therefore behave more positively. This leads to a positive outcome that includes decreased indexes of anxiety, such as depression [60]. Older adults have generally higher depression than other age groups [61]. That aging anxiety can be reduced through physical activity is especially important for pre-older adults.

Anxiety regarding aging is also a psychological variable that is closely related to physical activity. In this study, it was found that anxiety regarding aging had a negative effect on pre-older adults who engaged in high physical activity. Research has indicated that people who are not anxious about aging and think positively have a lifespan 7.5 years longer than those who express such anxiety. This suggests that physical activity should increase with aging [56,62,63].

Therefore, given the results of this study, it appears that Korean pre-older adults are thoroughly prepared for aging. Pre-older adults practically experienced the reality of current older adults in Korean society. Such an experience may have promoted aging-related anxiety in pre-older adults. The thought that they might experience the aging-related issues of their parents might have generated more active preparation and planning.

Second, a positive (+) relationship between physical activity and social support was found. When humans retire, they become less active. In such an environment, interpersonal relationships naturally decrease and the support available from non-family decreases markedly. However, practicing physical activity can change the individual’s environment [64]. The literature also suggests that physical activity or a healthy body positively correlates with social support [65].

Physical activity usually implies activities in different groups. Participating in exercises facilitates interaction with others and relieves stress. Further, when joining a group, one needs to establish a positive relationship with group members. To maintain such an activity, a country should encourage individuals to share ideas with others, since understanding others facilitates dialogue and these activities eventually lead to social support.

Since older adults decrease social activities after retirement, social environments and conditions for sustaining these activities must be created; however, it appears that Korean older adults are actively preparing for these activities. This could be because they enjoy various physical activities together with pre-older adults. At the present time, when life expectancy is increasing worldwide, individuals may continue to engage in physical activity as they age. In addition, various social activities and active interpersonal relationships are expected to continue to expand.

The current results indicated that social support had a negative effect (−) on aging anxiety. This indicates that when social support is positive anxiety about aging does not exert a significant effect. In particular, many studies have shown that psychological variables, such as depression and anxiety, are less prevalent when social support is high [66,67,68]. In this study, anxiety regarding aging was negative (−) among older Korean adults due to their engagement in various social activities and good interpersonal relationships.

Pre-older adults are still engaged in economic activity. Feelings of anxiety about aging may be psychologically uncomfortable and thereby have a negative (−) effect. In addition, pre-older adults are a group that has sustained economic activity for an extended period. That is, such persons are in a stable position in society. Socially stable positions can impact social recognition. Such a situation could be responsible for the negative results regarding aging anxiety.

Current older adults in Korea also participated in the workforce for an extended period. However, they were not socially recognized for their economic contribution. It is conceivable that social structure will change so that existing older adults maintain social activities and achieve mental stability. Moreover, not only the existing older adults but also the pre-older adults who will face such an environment in the future must have stable social activities and mental stability. This is a fundamental matter in Korean society, which is rapidly becoming an aging society.

Corroborating previous studies, social support played a mediating role between physical activity and aging anxiety. Social support shows how living in a community is valuable, especially for older adults; since various social activities have a positive effect on physical and mental health, efforts should be made to maintain various active pursuits.

Korea is becoming an aging society [69], and a large population of older adults already exists. However, in Korean society, it is difficult for the existing older adults to perform social activities comfortably and stably. Cultural factors also hinder social engagement; various social system factors in society limit the activities of existing older adults. Efforts by government and individuals are needed to improve awareness of this issue and to improve culture. A healthier Korean society could subsequently develop.

The study was conducted in the context of sociocultural characteristics that only appear in Korea’s prospective senior citizens, which accordingly differ from those in other countries. Therefore, it is not possible to generalize the current findings to all elderly persons worldwide. As such, it is necessary to investigate various elderly and prospective elderly populations and determine the relationships among relevant variables. IPAQ is a questionnaire that measures the amount of physical activity. However, it can be difficult to accurately measure human physical activity [70]. As such, further research should consider ways to measure the amount of physical activity of the elderly objectively and accurately. Qualitative research is also needed, based on in-depth interviews, which can reveal the experiences and needs of older persons.

This study indicates the current status of the nation’s prospective elderly, in a society on the verge of becoming an aging society. Accordingly, one can understand sociocultural aspects of the Korean prospective elderly and present policy alternatives for the current elderly that consider Korea’s transition to an aging society. In addition, the current work helps predict changes in Korea, whereby new cultures will emerge due to the aging society, and indicates positive directions in which culture might change. Moreover, the data may help relieve the fear of aging by providing indications of how persons may lead healthier lives. The work can be the basis for introducing programs to encourage physical activity among older persons and encouraging active social activity, so as to increase social support.

## 5. Conclusions

The study targeted prospective elderly people, namely the post-Korean War generation. The results indicated how the amount of physical activity and social support of Korean prospective seniors affected their aging anxiety and how social support is a parameter related to physical activity and aging anxiety. Prior studies have shown that high levels of physical activity in older adults increase social support and decrease fear of aging anxiety and anxiety [69,70]. These findings are consistent with those of the current study. However, there are few studies of how social support affects aging anxiety among older persons. The available studies indicate that social support and lifestyle have a positive effect on the health of the elderly [71,72]. The current study was limited to the elderly in Korea. Unlike other countries, the elderly in Korea have a high suicide rate, isolation, low subjective health levels, and the longest participation in labor. Hence, the role of social support in decreasing aging anxiety is especially meaningful for this group. Prospective elderly Koreans appear to be attempting to live a healthy life in preparation for aging, and are investing in themselves rather than in their children. Based on these results, further study of the elderly in Korea will help predict the structure of Korean society in the future.

## Figures and Tables

**Table 1:** Physical activity scoring.

	Physical Activity Scoring
Walking	MET-min/week = 3.3 × min of activity/day × days per week
Moderate PA	MET-min/week = 4.0 × min of activity/day × days per week
Vigorous PA	MET-min/week = 8.0 × min of activity/day × days per week
Total amount of PA	Walking MET-min/week + Moderate PA MET-min/week + Vigorous PA MET-min/week

PA: physical activity; MET: metabolic equivalent task.

**Table 2:** Exploratory factor analysis of social support.

Question	A Specific Person	The Family	A Friend
Item 3	0.972	0.042	−0.066
Item 2	0.956	−0.013	−0.001
Item 1	0.881	−0.011	0.017
Item 4	0.819	0.006	0.086
Item 11	0.053	0.930	−0.039
Item 12	−0.037	0.922	−0.023
Item 10	0.000	0.828	−0.008
Item 9	0.054	0.687	0.180
Item 8	−0.030	0.051	0.892
Item 6	0.028	−0.028	0.884
Item 7	−0.024	0.069	0.856
Item 5	0.067	−0.058	0.853
Eigenvalue	7.530	1.322	0.723
Variance explained (%)	62.751	11.021	6.024
Cumulative variance explained (%)	62.751	73.771	79.796
Cronbach’s *α*	0.955	0.935	0.925

KMO = 0.922, Bartlett = 9875.131, df = 66.000, *p* < 0.001. KMO: Kaiser-Meyer-Olkin

**Table 3:** Exploratory factor analysis of aging anxiety.

Question	Fear of Losses	Psychological Concerns	Fear of Old People	Physical Appearance
Item 18	0.868	0.049	0.042	0.020
Item 11	0.672	0.109	0.122	0.005
Item 16	0.527	0.047	0.051	0.198
Item 5	0.011	0.809	0.047	0.043
Item 17	0.064	0.718	0.010	0.033
Item 8	0.032	0.696	0.028	0.077
Item 14	0.034	0.664	0.065	0.032
Item 2	0.042	0.661	0.035	0.018
Item 6	0.006	0.568	0.074	0.002
Item 20	0.099	0.431	0.066	0.403
Item 10	0.008	0.009	0.847	0.022
Item 1	0.036	0.039	0.759	0.027
Item 13	0.043	0.010	0.716	0.151
Item 3	0.003	0.038	0.696	0.026
Item 19	0.350	0.016	0.486	0.042
Item 15	0.089	0.084	0.064	0.824
Item 12	0.127	0.072	0.089	0.621
Item 9	0.226	0.177	0.150	0.362
Eigenvalue	4.814	3.024	1.014	0.680
Variance explained (%)	26.745	16.799	5.634	3.779
Cumulative variance explained (%)	26.745	43.543	49.177	52.956
Cronbach’s *α*	0.764	0.851	0.846	0.778

KMO = 0.882, Bartlett = 5813.472, df = 153.000, *p <* 0.001. KMO: Kaiser-Meyer-Olkin

**Table 4:** Average variance extracted (AVE) and concept reliability (CR) for social support.

Section	A Specific Person	The Family	A Friend
Concept Reliability (CR)	0.955	0.935	0.925
Average Variance Extracted (AVE)	0.843	0.782	0.757

**Table 5:** AVE and CR for aging anxiety.

Section	Expectations of Aging	Social Worthlessness	Physical Weakness	Change in Appearance
Concept Reliability (CR)	0.775	0.844	0.830	0.782
Average Variance Extracted (AVE)	0.539	0.477	0.551	0.54

**Table 6:** Correlations between variables.

	Physical Activity	Social Support	Aging Anxiety
Physical Activity	1		
Social Support	0.162 ***	1	
Aging Anxiety	0.200 ***	0.432 ***	1

*** *p* < 0.001.

**Table 7:** Path coefficient of full-effects model.

Independent Variable		Dependent Variable	Estimate	SE	CR
Physical Activity	→	Aging Anxiety	−0.238	0.000	−5.130 ***

*** *p* < 0.001; SE: Scalar estimates; CR: Construct reliability.

**Table 8:** Path coefficients of mediation model.

	Independent Variable		Dependent Variable	Estimates	SE	CR
H1	Physical Activity	→	Aging Anxiety	−0.024	0.010	−2.289 *
H2	Physical Activity	→	Social Support	0.260	0.046	5.590 ***
H3	Social Support	→	Aging Anxiety	−0.130	0.017	−7.849 ***

* p < 0.05, *** *p* < 0.001; SE: Scalar estimates; CR: Construct reliability.

**Table 9:** Total effect, direct effect, and indirect effect.

Section	Total Effect (Direct Effects, Indirect Effects)		
	Physical Activity	Social Support	Aging Anxiety
Social Support	0.211 (0.211, 0.000)	0.000 (0.000, 0.000)	0.000 (0.000, 0.000)
Aging Anxiety	−0.207 (−0.086, −0.121)	−0.575 (−0.575, 0.000)	0.000 (0.000, 0.000)

**Table 10:** Statistical significance of mediating effect.

95% CI					
Physical Activity	→	Aging Anxiety	Lower	Upper	*p*
			−0.171	−0.078	0.004

**Table 11:** Path coefficients of mediated model.

	χ^2^	df	TLI	RMSEA (90% CI)
Partially mediated model	145.301	19	0.905	0.092 (−0.161, −0.083)
Fully mediated model	139.802	18	0.904	0.093 (−0.175, −0.091)

χ^2^ = 5.499, df = 1.
